# Hospital Admissions Secondary to Diseases of the Blood, Blood-Forming Organs, and Immune System in England and Wales

**DOI:** 10.7759/cureus.30179

**Published:** 2022-10-11

**Authors:** Moaath K Mustafa Ali, Abdallah Y Naser, Amal AbuAlhommos, Tamara Al-Daghastani, Hamzeh Alrawashdeh, Saja Mustafa Ali, Hassan Alwafi, Mohammed Mansour Alqurashi, Abdulaziz H Basha Ahmed, Hussein Albarqi

**Affiliations:** 1 Hematology and Medical Oncology/Taussig Cancer Institute, Cleveland Clinic Cancer Center, Cleveland, USA; 2 Applied Pharmaceutical Sciences and Clinical Pharmacy, Isra University, Faculty of Pharmacoepidemiology, Amman, JOR; 3 Pharmacy Practice, King Faisal University, Alhasa, SAU; 4 Medical Allied Sciences, Al-Balqa Applied University, As-Salt, JOR; 5 Ophthalmology, Sharif Eye Centers, Irbid, JOR; 6 Internal Medicine, Saint Agnes Hospital, Baltimore, USA; 7 Medicine, Umm Al-Qura University, Faculty of Medicine, Makkah, SAU; 8 Therapeutics and Toxicology, Al Noor Specialist Hospital, Ministry of Health, Makkah, SAU; 9 Intensive Care Unit, Ajyad Emergency Hospital, Ministry of Health, Makkah, SAU; 10 Internal Medicine, Al Noor Specialist Hospital, Ministry of Health, Makkah, SAU

**Keywords:** wales, england, hospital admissions, immune, blood

## Abstract

Background

Non-malignant hematologic and immune disorders-related hospitalization trends are unstudied despite their importance from a public health standpoint. Therefore, this study aimed to define the hospitalization trends of the International Statistical Classification of Diseases-10 (ICD-10) category diseases of the blood and blood-forming organs and certain disorders involving the immune mechanism (B&ID).

Methods

We conducted an ecologic study to analyze hospital admission data obtained from England's Hospital Episode Statistics database and Wales' Patient Episode Database. Hospital admissions data for non-malignant hematologic disorders and immune disorders were extracted for the period from April 1999 to March 2019. We used the Poisson model to assess trends in hospital admissions.

Results

The total annual B&ID-related hospital admission (RHA) rate for all categories increased by 137.9% between 1999 and 2019 (p<0.01). Females accounted for 54% of all B&ID-RHA. Around 37% of B&ID-RHA were seen in the age group of 15-59 years and 29% in the age group of 75 years and above. The most common causes of B&ID-RHA were aplastic and other anemias and other bone marrow failure syndromes, ICD-10 category (33%) and nutritional anemias category (28%). Certain disorders involving the immune mechanism category accounted for the least number of B&ID-RHA (8.4%). The highest increase in B&ID-RHA was seen in the nutritional anemias category (3.86 fold), followed by certain disorders involving the immune mechanism (1.28 fold). Iron deficiency anemia accounted for 95.1% of all hospitalizations secondary to nutritional anemias. Around half of all, hemolytic anemia category hospitalizations were secondary to the sickle cell anemia subcategory.

Conclusions

Hospital admissions trends in non-malignant hematologic and immune disorders changed dynamically among age groups and gender in England and Wales over the last two decades. Understanding these changes has important implications for public health planning.

## Introduction

The non-malignant hematologic diseases comprise an extensive range of disorders, including nutritional anemias, hemolytic anemias, aplastic anemias, bone marrow failure disorders, white blood cell disorders, spleen-related disorders, coagulation, and hemorrhagic disorders. These diseases result in a significant economic burden. In 31 European countries, the cost was estimated to be €11 billion in 2012, comparable to the cost of malignant hematologic diseases [1]. However, despite the significant economic impact, only 2.2% of the European health research budget was allocated to hematologic diseases between 2007 and 2013 [2]. In the United Kingdom (UK), the estimated healthcare cost secondary to non-malignant hematologic diseases per 10 citizens was €154 [1].

Disorders of the immune system include both autoimmune disorders and immunodeficiency disorders. Primary immune deficiencies result from defects in immune system function and development. These disorders are primarily chronic and result in recurrent infections [3]. The associated costs of treatments, diagnostics, and hospital admissions can be significant [4-6]. Moreover, these patients experience significant physical and psychological burdens [7,8].

There is a significant gap in understanding and measuring the health burden of non-malignant hematologic diseases or immune deficiencies worldwide. Moreover, there is a lack of studies investigating the trends of hospital admissions related to these diseases in Europe, including the United Kingdom. Herein, we conducted an ecologic study to define the trends of hospital admissions secondary to the International Statistical Classification of Diseases-10 (ICD-10) category diseases of the blood and blood-forming organs and certain disorders involving the immune mechanism (B&ID) in England and Wales between 1999 and 2019, using publicly available data.

## Materials and methods

Study sources and the population

As previously described [9-11], we conducted an ecological study using publicly available data extracted from the Hospital Episode Statistics (HES) database in England and the Patient Episode Database for Wales (PEDW) between April 1999 and April 2019 [12,13]. The HES and PEDW databases contain hospital admission data for patients with B&ID from all age groups, which are subdivided into four categories - below 15 years, 15-59 years, 60-74 years, and 75 years and above. We identified B&ID-related hospital admissions (B&ID-RHA) using the 10th version of the ICD system. All diagnostic codes for B&ID (D50-D89) were used to identify hospital admission related to these disorders in England and Wales. The HES and PEDW databases record all hospital admissions, outpatients, and accident and emergency activities performed at all National Health Service (NHS) trusts and any independent sector funded by NHS trusts. Data for hospital admissions in England and Wales are available from 1999 to 2000 onwards. Available data include patient demographics, clinical diagnoses, duration of stay, and procedures. The HES and PEDW data are checked regularly to ensure their validity and accuracy [12,13]. To calculate the yearly hospital admission rate for all diseases, we collected midyear population size data from 1999 to 2019 from the Office for National Statistics [14]. The annual rate was estimated by dividing the number of admissions by the number of populations of interest.

Statistical analysis

Hospital admission rates with 95% confidence intervals (CIs) were calculated using the finished consultant episodes of B&ID-related hospital admission (RHA) divided by the midyear population. We used the chi-squared test to assess the difference between the hospital admission rates between 1999 and 2019. All analyses were conducted using SPSS version 25 (IBM Corp, Armonk, NY).

## Results

A total number of 5,803,821 hospital admissions were recorded during the study period. The total annual absolute number for B&ID-RHA for different causes increased by 171.2% from 168,577 in 1999 to 457,254 in 2019. Similarly, the B&ID-RHA rate increased by 137.9% from 323.32 (95% CI 321.77-324.86) in 1999 to 769.27 (95% CI 767.05-771.49) in 2019 per 100,000 persons (trend test, p<0.01).

The causes of B&ID-RHA are described in Table 1. There was no hospital admission in England and Wales for D78-D78 "intraoperative and postprocedural complications of the spleen" identified in the database during the study duration.

**Table 1:** Percentage of ICD-10 category diseases of the blood and blood-forming organs and certain disorders involving the immune mechanism hospital admission from the total number of admissions. ICD: International Statistical Classification of Diseases

ICD code	Description	Percentage of total number of admissions
D50-D53	Nutritional anemias	28.4%
D55-D59	Hemolytic anemias	12.4%
D60-D64	Aplastic and other anemias and other bone marrow failure syndromes	33.1%
D65-D69	Coagulation defects, purpura, and other hemorrhagic conditions	8.9%
D70-D77	Other disorders of blood and blood-forming organs	8.7%
D80-D89	Certain disorders involving the immune mechanism	8.4%

**Figure 1:**
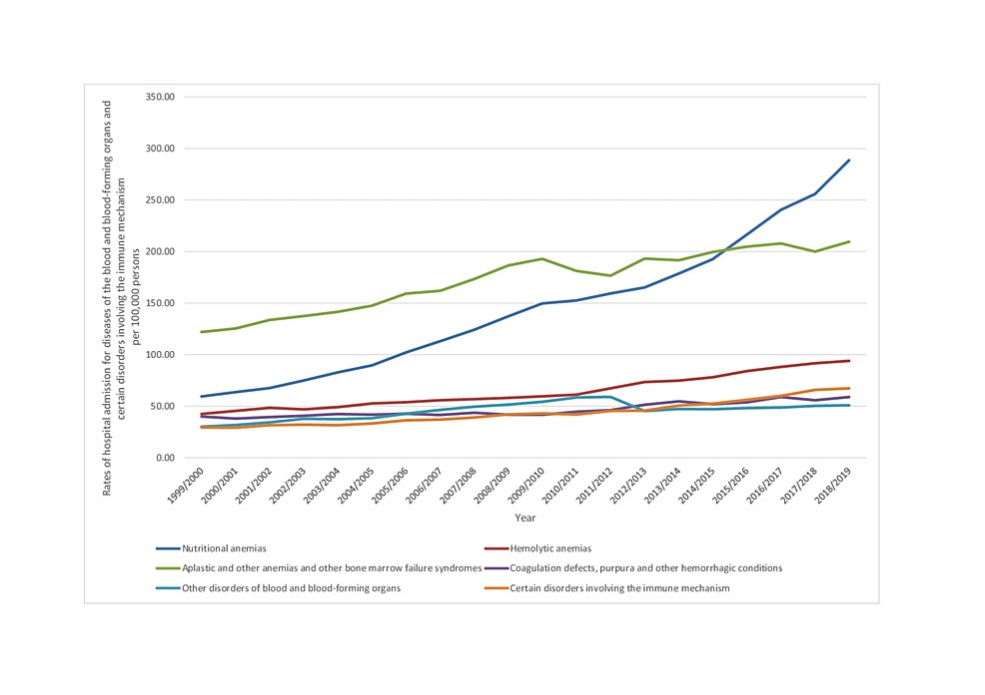
Rates of hospital admission for ICD-10 category diseases of the blood and blood-forming organs and certain disorders involving the immune mechanism in England and Wales stratified by type between 1999 and 2019. ICD: International Statistical Classification of Diseases

During the past two decades, the rate of B&ID-RHA increased in most of the categories. The highest increase in rate was observed in the following ICD-10 categories: nutritional anemias (3.86 fold), certain disorders involving the immune mechanism (1.28 fold), and hemolytic anemias (1.22 fold) (p<0.01) (Table 2, Figure 1).

**Table 2:** Percentage change in the hospital admission rates for ICD-10 category diseases of the blood and blood-forming organs and certain disorders involving the immune mechanism from 1999 to 2019 in England and Wales. ICD: International Statistical Classification of Diseases

Diseases	Rate of diseases in 1999 per 100,000 persons (95% CI)	Rate of diseases in 2019 per 100,000 persons (95% CI)	Percentage change from 1999 to 2019
Nutritional anemias	59.45 (58.79-60.11)	288.63 (287.27-290.00)	385.5%
Hemolytic anemias	42.43 (41.87-42.99)	93.97 (93.20-94.75)	121.5%
Aplastic and other anemias and other bone marrow failure syndromes	121.90 (120.96-122.85)	209.53 (208.37-210.70)	71.9%
Coagulation defects, purpura, and other hemorrhagic conditions	39.98 (39.44-40.53)	58.90 (58.28-59.52)	47.3%
Other disorders of blood and blood-forming organs	30.09 (29.62-30.56)	50.94 (50.37-51.52)	69.3%
Certain disorders involving the immune mechanism	29.46 (29.00-29.93)	67.29 (66.63-67.95)	128.4%

Iron deficiency anemia subcategory (D50) accounted for 95.1% of all hospitalizations secondary to nutritional anemias category, followed by folate deficiency anemia subcategory (D52), which accounted for 2.7% of all nutritional anemia category hospitalizations. The hospitalization rate for iron deficiency anemia has increased by 4.22 folds and 1.85 folds for folate deficiency anemia. In the ICD-10 category, aplastic and other anemias and other bone marrow failure syndromes (D60-D64), other anemias subcategory (D64) and other aplastic anemias and other bone marrow failure syndromes subcategory (D61) accounted for 81.4-12.9% of all admissions. In hemolytic anemias (D55-D59), sickle cell disorders (D57) accounted for 50.2% of admissions, thalassemia (D56) accounted for 31.9%, and acquired hemolytic anemias (D59) accounted for 13.9%. Over the last two decades, the hospitalization rate for sickle cell disorders increased by 1.95 folds. In the ICD-10 category, certain disorders involving the immune mechanism (D80-D89), the subcategory immunodeficiency with predominantly antibody defects (D80) accounted for 42% of admissions, followed by common variable immunodeficiency (D83), which accounted for 22.6% of admissions, followed by sarcoidosis (D86), which accounted for 12.3% of admissions. The rate of hospital admissions increased in common variable immunodeficiency, immunodeficiency with predominantly antibody defects, and sarcoidosis by 3.45 fold, 1.13 fold, and 1.04 fold, respectively. In the ICD-10 category of coagulation defects, purpura, and other hemorrhagic conditions (D65-D69), the most common causes of admission were purpura and other hemorrhagic conditions (D69) (72.4%), hereditary factor VIII deficiency (D66) (12.6%), and other coagulation defects (D68) (12.4%) subcategories. Admissions due to hereditary factor IX deficiency accounted only for 2.2% of admissions. The hospitalization rate for purpura and other hemorrhagic conditions subcategory (D69) has increased by 1.04 folds. On the other hand, the rate of hospitalization decreased in the following subcategories: hereditary factor IX deficiency (D67) (47.7%), disseminated intravascular coagulation (D65) (36%), hereditary factor VIII deficiency (D66) (30.4%), and other coagulation defects (D68) (26.1%).

**Figure 2:**
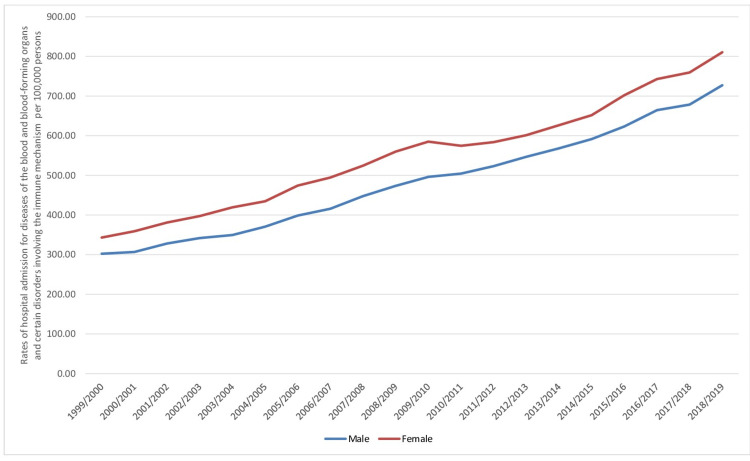
Rates of hospital admissions for ICD-10 category diseases of the blood and blood-forming organs and certain disorders involving the immune mechanism in England and Wales stratified by gender between 1999 and 2019. ICD: International Statistical Classification of Diseases

A total of 5,803,821 B&ID-RHA occurred in England and Wales throughout the study duration. Females contributed to 3,137,256 B&ID-RHA, accounting for 54.1% of the total admissions and averaging 156,862 admissions per year. In males, the rate of B&ID-RHA increased by 140.5% from 302.39 (95% CI 300.26-304.53) in 1999 to 727.19 (95% CI 724.12-730.27) in 2019 per 100,000 persons. On the other hand, the B&ID-RHA rate increased by 136.1% from 343.23 (95% CI 341.01-345.45) in 1999 to 810.37 (95% CI 807.17-813.58) in 2019 per 100,000 persons (Figure 2).

**Figure 3:**
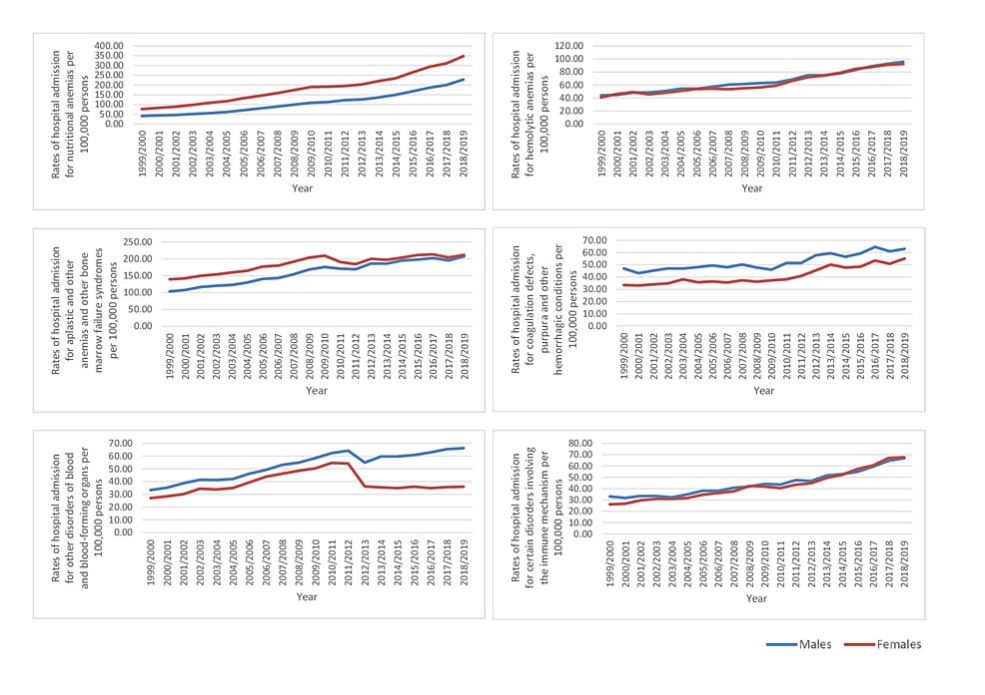
Hospital admission rates for ICD-10 category diseases of the blood and blood-forming organs and certain disorders involving the immune mechanism in England and Wales stratified by gender between 1999 and 2019. ICD: International Statistical Classification of Diseases

The B&ID-RHA rates for ICD-10 categories of hemolytic anemias, coagulation defects, purpura, and other hemorrhagic conditions, other disorders of blood and blood-forming organs, and certain disorders involving the immune mechanism were higher in males compared to females. On the other hand, the rates of B&ID-RHA for ICD-10 categories nutritional anemias, aplastic, and other anemias and other bone marrow failure syndromes were higher in females (p<0.05) (Figure 3).

**Figure 4:**
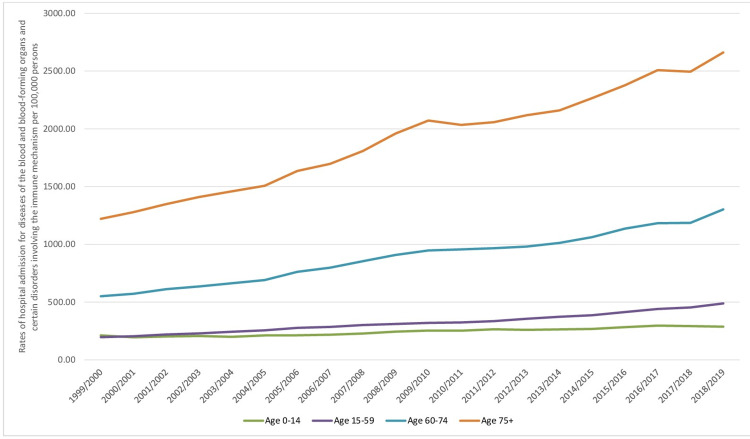
Hospital admission rates for ICD-10 category diseases of the blood and blood-forming organs and certain disorders involving the immune mechanism in England and Wales stratified by age group between 1999 and 2019. ICD: International Statistical Classification of Diseases

Regarding age group distribution for B&ID-RHA, the age group 15-59 years accounted for 37.0% of the total number of hospital admissions, followed by the age group 75 years and above with 29.4%, the age group 60-74 years with 25.2%, and then the age group below 15 years with 8.4%. The rate of B&ID-RHA among patients aged below 15 years increased by 35.7% from 211.75 (95% CI 208.88-214.61) in 1999 to 287.43 (95% CI 284.23-290.64) in 2019 per 100,000 persons. Rates of B&ID-RHA among patients aged 15-59 years increased by 148.8% from 196.27 (95% CI 194.72-197.81) in 1999 to 488.26 (95% CI 485.93-490.59) in 2019 per 100,000 persons. The rate of B&ID-RHA among patients aged 60-74 years increased by 136.7% from 550.53 (95% CI 545.03-556.04) in 1999 to 1302.94 (95% CI 1295.63-1310.25) in 2019 per 100,000 persons. The rate of B&ID-RHA among patients aged 75 years and above increased by 118.1% from 1220.47 (95% CI 1209.59-1231.35) in 1999 to 2661.25 (95% CI 2647.25-2675.24) in 2019 per 100,000 persons (Figure 4).

**Figure 5:**
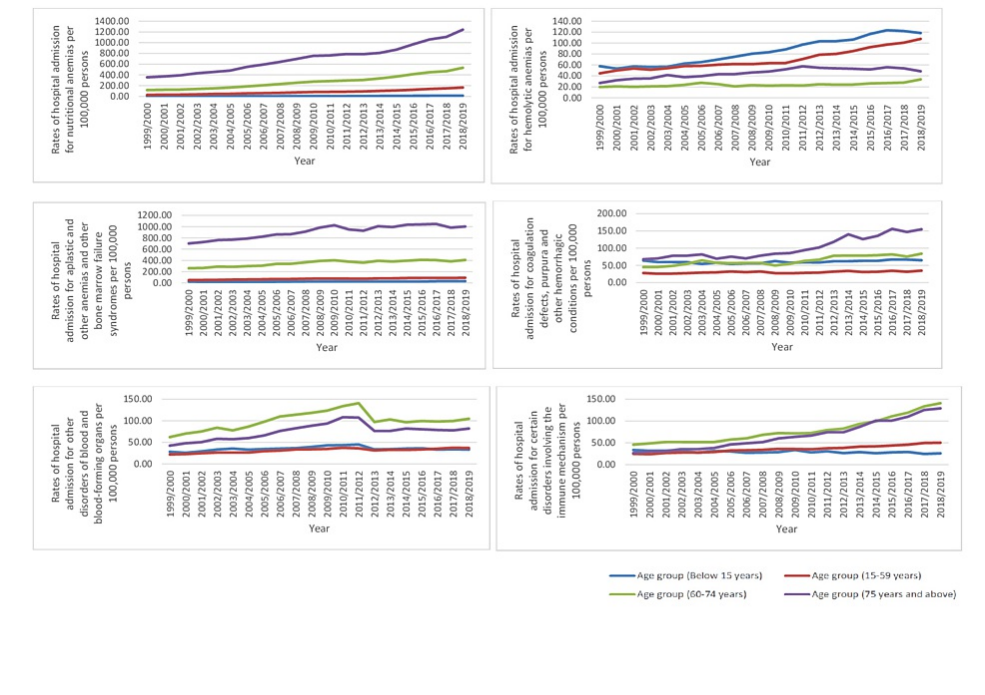
Hospital admission rates for ICD-10 category diseases of the blood and blood-forming organs and certain disorders involving the immune mechanism in England and Wales stratified by age group between 1999 and 2019. ICD: International Statistical Classification of Diseases

The hospital admission for ICD-10 categories coagulation defects, purpura, and other hemorrhagic conditions were more prevalent in the age groups: 75 years and above, followed by 60-74 years, followed by below 15 years, and lastly by 15-59 years, respectively. In addition, the admissions for ICD-10 category certain disorders involving the immune mechanism were higher among the age group: 60-74 years, 75 years and above, 15-59 years, and lastly, below 15 years. Furthermore, the hospital admissions for ICD-10 category, other disorders of blood and blood-forming organs were higher in the age groups: 60-74 years, 75 years and above, below 15 years, and 15-59 years. Lastly, hospital admissions for ICD-10 category, hemolytic anemias were higher in the age group: below 15 years, 15-59 years, 75 years and above, and 60-74 years (Figure 5).

## Discussion

This study describes hospital admissions trends in B&ID, which are primarily unstudied. Our study showed a 171% increase in B&ID-RHA in England and Wales over the last two decades. The UK population increased from 58.8 million in 1999 to 66.7 million in 2019, representing a 13% increase [14]. However, this population size increase cannot solely explain the increase in admissions rates. Both 60-74 and 75 years and above age groups accounted for more than half of hospital admissions. In the United Kingdom, the population structure continues to change. In 2016, 11.8 million UK residents were 65 years and above, representing 18% of the population [15]. The elderly are more likely to have many chronic comorbidities and are at higher risk for hospital admissions than younger age groups [15]. In 2006, the percentage of NHS hospital admissions for 66 years and above was 36% and increased to 41% in 2016 [15]. Our study showed that more than half of admissions occurred at 60 years and above age. Furthermore, these age groups experienced a more than 100% increase in hospitalization rates. 

Hospital admissions related to ICD-10 category, aplastic and other anemias, and other bone marrow failure syndromes (D60-D64) accounted for one-third of all admissions. The subcategory, other anemias (D64) was the predominant cause of admission (80%) in this group and includes a large group of heterogenous anemias; some are unspecified. Moreover, anemia due to chronic diseases and antineoplastic chemotherapy are listed under other anemias subcategory (D64). Anemia of chronic diseases is the second most common cause of anemia worldwide [16]. The accurate determination of the prevalence of anemia in chronic diseases is difficult because it is commonly a diagnosis of exclusion and is confused with iron deficiency anemia [17]. The incidence of anemia in chronic diseases increases with aging, affecting 77% of the elderly, in whom no clear diagnosis is determined easily [17].

The second most common cause of admissions was nutritional anemias (D50-D53), accounting for 28% of all admissions in England and Wales. Nutrition anemia has experienced the fastest increase in the rate of hospitalization during the last two decades. The predominant cause of admissions in this category was iron deficiency anemia (95%). Moreover, iron deficiency anemia-related admissions experienced a 4.2-fold increase in hospital admission rates. Iron deficiency anemia is the most prevalent anemia worldwide and the number one cause of anemia burden globally [18]. Iron deficiency anemia affects between 4 and 6 billion people globally [19]. In the English Longitudinal Study of Ageing, 8.8% of 4,451 elderly were found to have non-anemic iron deficiency, which was more common in females (10.9%) than in males (6.35%) [20]. The UK's National Diet and Nutrition Survey showed that the prevalence of iron deficiency anemia in girls aged 11-19 years was 9%, compared to 5% in the previous survey [21]. Moreover, the survey found that iron deficiency was higher in females [21]. The higher prevalence of iron deficiency in females partly explains the higher rate of iron deficiency anemia-related hospitalizations in females than males observed in our study.

Hemolytic anemia was the third most common cause of B&ID-RHA and accounted for 12% of admissions. Half of the hemolytic anemia-related admissions were from sickle cell disorders, and one-third were due to thalassemia. Sickle cell disorder admission rate increased by 1.95 folds, consistent with previous reports [22]. The increase in admission rate for sickle cell disorders is likely explained, at least partly, by the improvement in the life expectancy of patients with sickle cell disorders. The survival of patients with sickle cell disorder in high-income countries in the last 60 years has improved remarkably [23]. In the 1960s, it was described as a childhood disease, but nowadays, 99% of children with sickle cell disorder reach adulthood [23,24]. The improvement in survival increases the pool of patients who are susceptible to hospitalization. Moreover, with aging, patients with sickle cell disorder develop disease-related complications, resulting in increased hospitalization. Similarly, the survival of patients with thalassemia has improved significantly in the United Kingdom, attributed to the increased utilization of bone marrow transplants in children and iron chelation in adults [25]. In the United Kingdom, the life expectancy of patients with thalassemia increased from 17 years in 1970 to more than 40 years in 2000 [25]. The migration of people from sickle cell and thalassemia high prevalence areas to the United Kingdom has also increased the prevalence of these disorders [26]. Because of the limited life expectancy of patients with thalassemia and sickle cell anemia compared to the general population, hospitalizations were primarily seen in age groups below 15 years and 15-59 years. Our study showed that the hospitalization rate secondary to anemias due to enzyme disorders was higher in males. Glucose-6-phosphate dehydrogenase deficiency, which is the most prevalent enzyme deficiency globally, is predominantly a male disorder [27].

Coagulation defects, purpura, and other hemorrhagic conditions-related hospitalizations accounted for 8.9% of total B&ID-RHA in England and Wales. Three-quarters were related to subcategory purpura and other hemorrhagic conditions (D69), which consist mainly of platelet disorders. Thrombotic thrombocytopenic purpura is listed under this category. Admissions related to hereditary factor VIII deficiency were 5.7 folds of factor IX deficiency. Admissions in these two disorders were mainly seen in males because they are inherited as X-lined recessive. The prevalence of hemophilia A (factor VIII deficiency) is reported at 17.1 per 100,000 males compared to hemophilia B (factor IX deficiency), which is 3.8 per 100,000 males [28]. Around 35% of hemophilia cases are severe compared to 28% in hemophilia B, which partly explains the higher admission rates observed in our study in hemophilia A than B [28]. Our study showed that hemophilia A and B hospitalizations have decreased, possibly related to improved care and earlier diagnosis [29]. Other coagulation defects (D68) accounted for 12.4% of admissions in this category. It includes a large heterogeneous group of disorders characterized by increased or decreased coagulation, acquired or hereditary (e.g., von Willebrand's disease, prothrombin gene mutation, and antiphospholipid syndrome).

The ICD-10 category of certain disorders involving the immune mechanism has contributed to the least number of B&ID-RHA (8.2%) in England and Wales over the last two decades. Immunodeficiency with predominantly antibody defects (D80) accounted for 42% of admissions. This subcategory includes hereditary and non-familial hypogammaglobulinemia, selective immunoglobulin deficiencies, and other unspecified categories. Common variable immunodeficiency (D83) was the second most common cause of admissions in this category and contributed to one-fifth of admissions. Common variable immunodeficiency is a large group of genetic disorders that result in severe IgG immunodeficiency combined with IgA or IgM deficiency. In primary immunodeficiency registries, common variable immunodeficiency is the most common diagnosis and is reported in 20% of patients registered [30,31]. The rate of hospital admissions increased in common variable immunodeficiency by 3.45 fold. This might be due to increasing awareness and testing of the disease. In our study, the increase was seen in age groups of more than 15 years. The diagnosis of common variable immunodeficiency occurs mainly with the onset of symptoms between 20 and 30 years old [32,33]. On the other hand, combined immunodeficiency admissions were mainly in the age group below 15 years. Combined immunodeficiencies are heterogeneous genetic disorders and mostly lead to significant immunodeficiency with increased infections during early childhood.

Our study has several limitations. It is based on aggregated data rather than single patient level. Hence, we could not explore the impact of other factors on the admission rate. Because of this, ecological studies are subject to confounding. Variations in disease recording quality can also affect data quality and the interpretation of our analysis. This study reported hospital admissions data may have overestimated the presented admission rates because it includes emergency, readmission, and elective admissions. Moreover, the variation in management of diseases from outpatient to inpatient and vice versa can affect hospitalization trends.

## Conclusions

The trend of admissions related to blood and immune disorders in England and Wales experienced dynamic changes. This could be driven by the growing population size, variation in disease prevalence, improved diagnostic tools, aging population, and migration. Therefore, it remains essential to understand these trends and changes in hospitalization rates from a public standpoint. Furthermore, despite the significant economic burden of non-malignant hematologic disorders in Europe and the United Kingdom, little is known about immune disorders' economic impact. Therefore, this study presents data on hospitalization trends for these two disorders in England and Wales and addresses part of the unstudied picture.
